# Hypercapnic Respiratory Failure Secondary to Myxedema Coma: A Case Report

**DOI:** 10.7759/cureus.109809

**Published:** 2026-05-28

**Authors:** Carrine S Kogulan, Oluwafemi Ajibola

**Affiliations:** 1 Internal Medicine, Kansas City University - Joplin, Joplin, USA; 2 Department of Medicine, Nuvance Health, Vassar Brothers Medical Center, Poughkeepsie, USA

**Keywords:** acute hypercapnic respiratory failure, critcal care, hypothyroid myxedema coma, respiratory support, thyroid pathology

## Abstract

Hypothyroidism affects a large subset of the population, with many undiagnosed. In iodine-sufficient regions, chronic autoimmune thyroiditis (Hashimoto’s) is the leading cause. Chronic, untreated hypothyroidism can result in multisystem complications, including cardiovascular disease, infertility, and respiratory failure. The most severe presentation, myxedema, is a medical emergency marked by altered mentation, ranging from lethargy to coma, hypothermia, and hypoventilation. Prompt recognition and treatment with thyroid hormone replacement are essential to improve outcomes. We present a case of hypercapnic respiratory failure secondary to myxedema coma. A 51-year-old female with morbid obesity (BMI 39.9 kg/m²) presented unresponsive, hypoxemic, and cyanotic, requiring emergent intubation. Labs revealed severe anemia (Hb 6.5 g/dL) and respiratory acidosis. The urinalysis was positive for infection, and the urine culture grew *Escherichia (E.) coli*. Imaging showed small pericardial and pleural effusions, and echocardiography revealed preserved ejection fraction (55%). Thyroid studies confirmed severe hypothyroidism with thyroid-stimulating hormone (TSH) 38.2 mIU/L and free thyroxine (T₄) < 0.07 ng/dL, consistent with myxedema coma. She was managed with IV levothyroxine, IV hydrocortisone, antibiotics, ventilatory support, diuresis, and electrolyte repletion. Following thyroid hormone therapy, her respiratory status and mentation improved, allowing extubation and discharge after 14 days on 2 L home oxygen.

Myxedema coma-induced hypercapnic respiratory failure occurs through reduced central respiratory drive, respiratory muscle weakness, and impaired gas exchange from pleural effusions or pneumonia. Studies have shown diminished maximal inspiratory and expiratory pressures in hypothyroid patients, reversible with hormone replacement. Pericardial effusion, observed in up to one-third of severe hypothyroid cases, results from increased capillary permeability and typically resolves with levothyroxine therapy without invasive intervention. Hypercapnic respiratory failure secondary to myxedema coma is rare but life-threatening. Early diagnosis and thyroid hormone replacement are essential to prevent morbidity and mortality. Clinical vigilance is required for patients with unexplained respiratory failure, particularly women with nonspecific hypothyroid symptoms. Ensuring medication adherence, thyroid function monitoring, and adequate respiratory support is vital for preventing recurrence and improving outcomes.

## Introduction

Overt hypothyroidism affects approximately 0.3-2%, whereas subclinical hypothyroidism affects 4-10% of the population in iodine-sufficient areas, with many undiagnosed [1]. Women are disproportionately affected due to a higher prevalence of autoimmune disease [2]. Globally, iodine deficiency is the most common cause, whereas chronic autoimmune thyroiditis (Hashimoto’s) predominates in iodine-sufficient regions. The diagnosis of hypothyroidism relies on clinical evaluation and confirmation with thyroid function tests, including thyroid-stimulating hormone (TSH) and free thyroxine (T₄). Symptoms of hypothyroidism include fatigue, weight gain, depression, brittle hair, cold intolerance, and cognitive impairment. Chronic, untreated hypothyroidism can slow metabolic processes and lead to multisystem complications such as cardiovascular disease, infertility, respiratory complications, and myxedema [2]. Prompt treatment with levothyroxine and monitoring of thyroid function can prevent these severe manifestations, including life-threatening myxedema coma. We present a case of hypercapnic respiratory failure secondary to myxedema coma.

## Case presentation

A 51-year-old female with morbid obesity (BMI 39.9 kg/m²) presented to the emergency department after being found unresponsive, hypoxemic, and cyanotic by EMS, with significant periorbital edema. Despite severe respiratory compromise, she had managed to call her daughter, who activated emergency services. On arrival, she was in hypoxemic respiratory failure, requiring emergent intubation and ICU admission.

**Figure 1:**
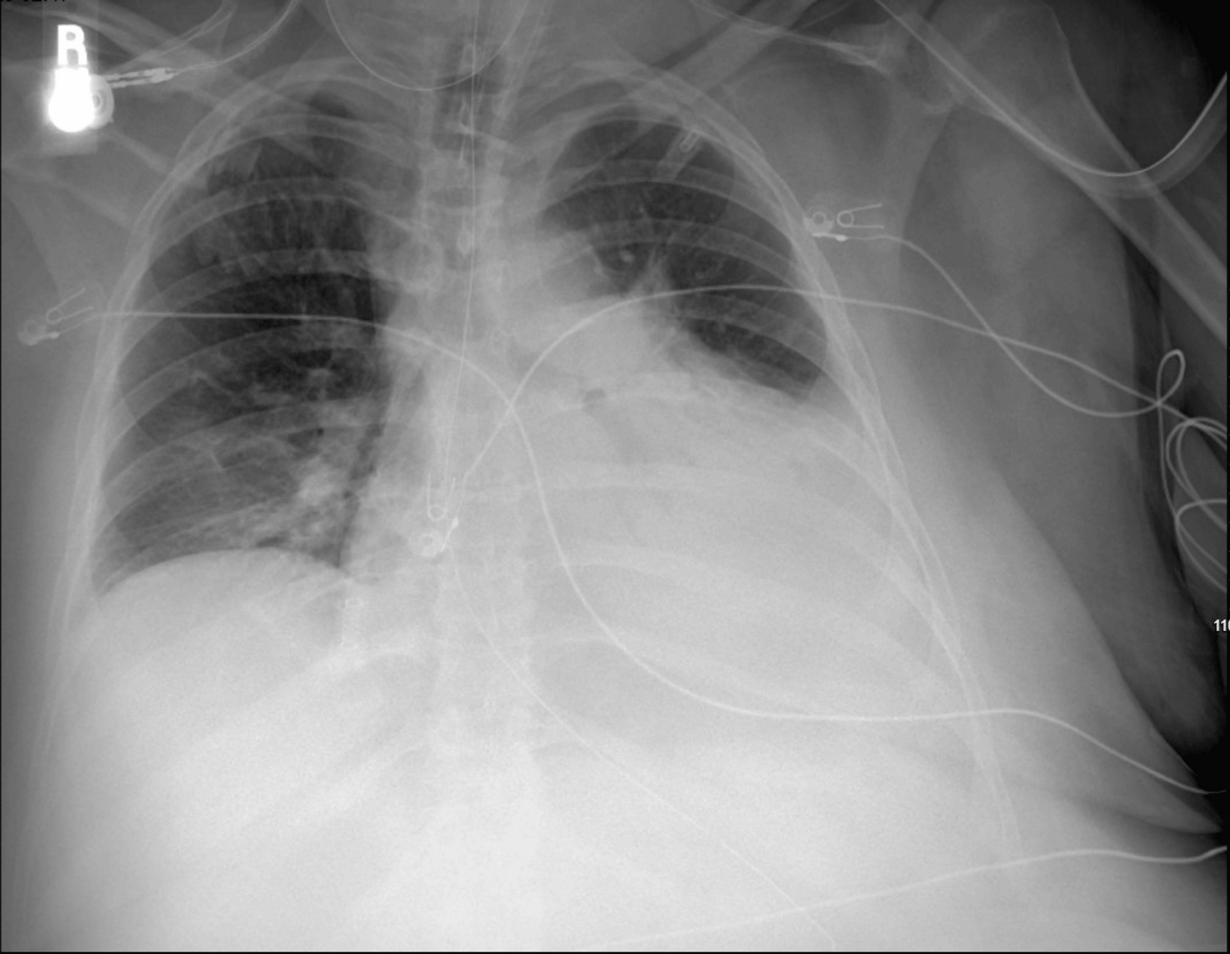
Initial chest X-ray imaging significant for bilateral pleural effusions and lower lobe infiltrates Portable chest radiograph obtained in the anteroposterior (AP) view.

Her initial laboratory evaluation (Table 1) revealed severe anemia with a hemoglobin of 6.5 g/dL, necessitating transfusion of two units of packed red blood cells, leukopenia (WBC 3.4×10³/μL), neutrophilia (79.3%), mild elevations in liver enzymes, elevated creatine kinase, and normal creatinine, blood urea nitrogen (BUN), prothrombin time (PT) 13.0 sec, and international normalized ratio (INR) 1.0. Her vital signs were significant for a temperature of 96.5 °F, blood pressure 91/54, pulse 58, and oxygen saturation 87%. The arterial blood gas taken on admission demonstrated post-hypercapnic metabolic alkalosis with compensatory respiratory acidosis. The urinalysis was positive for infection, and the urine culture grew *Escherichia (E.) coli*. The chest X-ray showed small bilateral pleural effusions and lower lobe infiltrates (Figure 1), while the brain CT was unremarkable. Transthoracic echocardiography revealed a preserved ejection fraction of 55% with a small pericardial effusion.

**Table 1:** Pertinent laboratory findings on admission AST = aspartate aminotransferase; ALT = alanine aminotransferase; BNP = B-type natriuretic peptide; BUN = blood urea nitrogen; HCO₃ = bicarbonate; pCO₂ = partial pressure of carbon dioxide; pO₂ = partial pressure of oxygen; WBC = white blood cell count; INR = international normalized ratio; PT = prothrombin time; TSH = thyroid-stimulating hormone; free T4 = free thyroxine

Lab	Patient Value	Normal Range
Hemoglobin	6.5 g/dL	12–16 g/dL
AST	70 U/L	10–40 U/L
ALT	36 U/L	7–56 U/L
BNP	234 pg/mL	<100 pg/mL
Creatinine	0.8 mg/dL	0.6–1.2 mg/dL
BUN	20 mg/dL	7-18 mg/dL
Creatine Kinase	224 U/L	38–174 U/L
HCO₃	47 mEq/L	22-28 mEq/L
pH	7.46	7.35-7.45
pCO₂	62.3 mmHg	33-45 mmHg
pO₂	46 mmHg	75-105 mmHg
WBC	3400/mm^3^	4500-11,000/mm^3^
Neutrophils	79.3%	54-62%
INR	1.0	0.8-1.1
PT	13.0 seconds	11-15 seconds
TSH	38.2 mIU/mL	0.4-4.0 mIU/mL
Free T4	<0.07 ng/dL	0.9-1.7 ng/dL

Thyroid function testing on hospital day 2 demonstrated severe hypothyroidism with a TSH of 38.2 mIU/L and free T₄ of <0.07 ng/dL. The delay in thyroid function testing was due to diagnostic uncertainty, as thyroid dysfunction was initially low on the differential, given the absence of a known history of thyroid disease. Diagnosis was established based on clinical presentation and supported by the Popoveniuc diagnostic scoring system [3]. The patient was treated for myxedema coma with an intravenous bolus of levothyroxine 250 mcg and hydrocortisone 100 mg, mechanical ventilatory support, diuresis for fluid overload, electrolyte repletion, and empiric intravenous antibiotics: initially, vancomycin and meropenem, later transitioned to ceftriaxone and azithromycin. Broad-spectrum antibiotics were initiated due to suspected sepsis, which was considered the precipitating factor for myxedema coma. Following therapy, the TSH improved to 14.1 mIU/L and free T₄ to 0.25 ng/dL by day 7.

**Figure 2:**
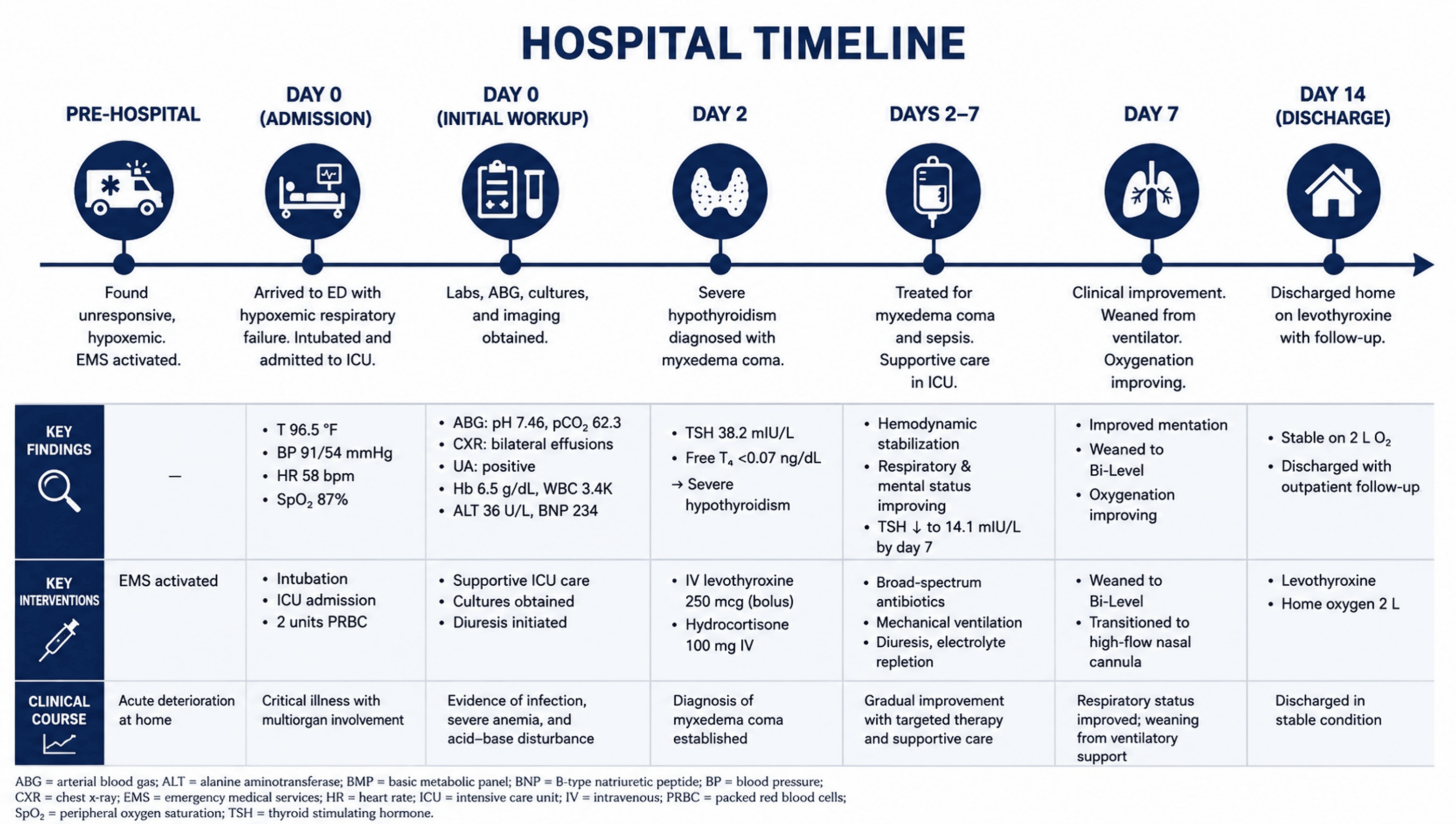
Hospital timeline highlighting significant events The figure was created using Canva (Canva Pty Ltd., Sydney, Australia).

Over the course of hospitalization (Figure 2), her mentation and respiratory function gradually improved, allowing for extubation to Bi-Level on day 7 and transition to high-flow nasal cannula. She was discharged after 14 days of hospitalization on 2 L of home oxygen with instructions for strict adherence to levothyroxine therapy and outpatient follow-up. Home oxygen was required at discharge due to persistent supplemental oxygen needs during recovery from hypoxemic respiratory failure. 

## Discussion

Myxedema coma is a life-threatening complication of severe, untreated hypothyroidism. It is often diagnosed clinically based on altered mental status, supportive laboratory findings, and evidence of severe hypothyroidism [4]. The syndrome typically presents with altered mental status, hypothermia, hemodynamic instability, dry skin, hoarse voice, delayed tendon reflexes, macroglossia, and nonpitting edema [5].

**Figure 3:**
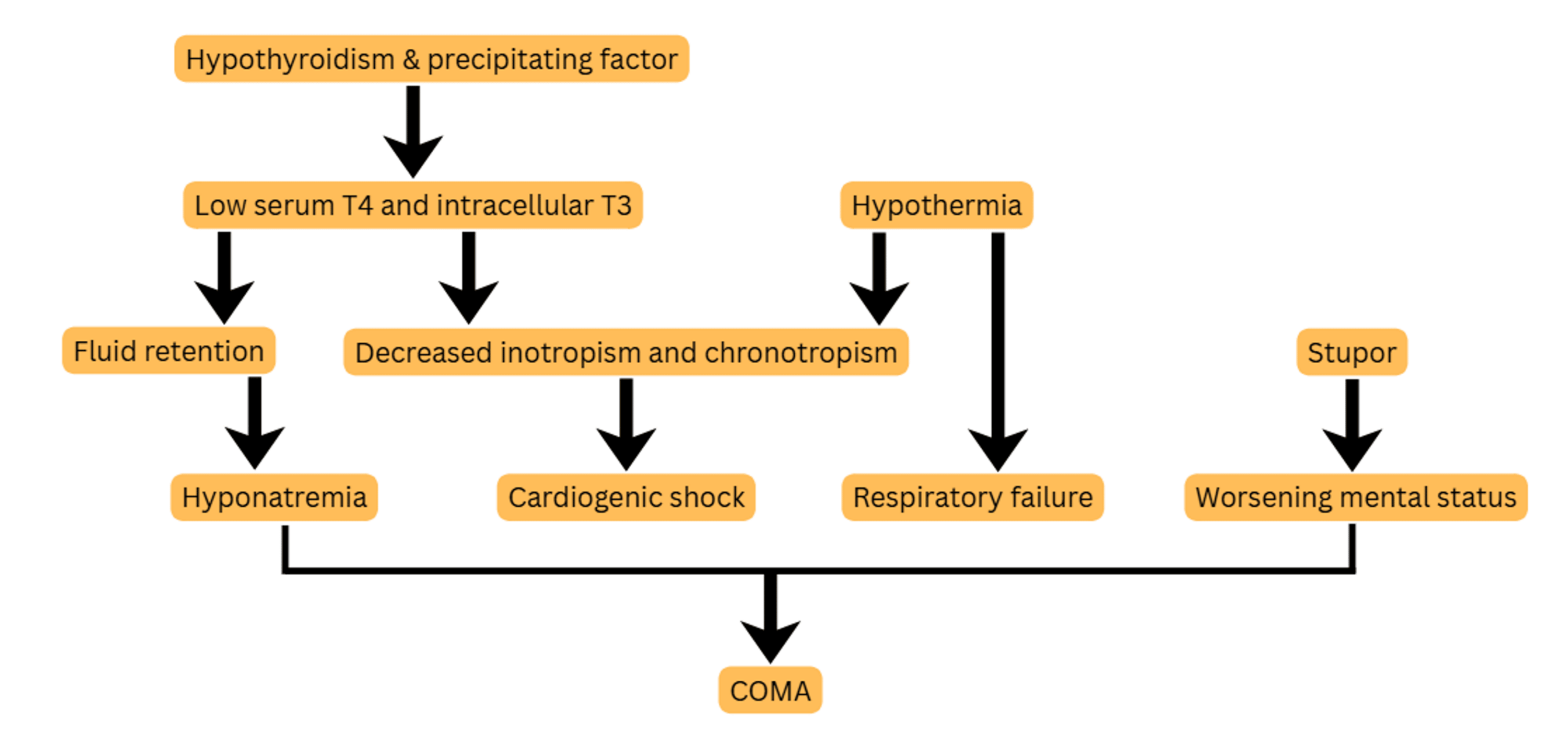
Pathogenesis of myxedema coma The figure was created using Canva (Canva Pty Ltd., Sydney, Australia).

Biochemically, profound deficiencies of T4 and triiodothyronine (T3) contribute to fluid retention, reduced cardiac inotropy and chronotropy, worsening mental status, and respiratory failure, all of which can progress to coma (Figure 3). Myxedema coma has also been associated with coagulation abnormalities and arrhythmias.

Respiratory failure in patients with myxedema coma is multifactorial, involving diminished central nervous system responsiveness to hypoxia and hypercapnia, respiratory muscle dysfunction, pleural effusions, reduced lung volumes, pneumonia, and aspiration [5].

Studies have shown that hypothyroid patients exhibit markedly reduced maximal inspiratory and expiratory pressures, which improve with thyroid hormone replacement, and respiratory myopathy may result from diaphragmatic weakness due to phrenic nerve involvement [6]. The degree of thyroid dysfunction is directly related to the amount of respiratory muscle weakness [6].

Additionally, pericardial effusions occur in approximately one-third of patients with severe hypothyroidism due to increased vascular permeability, although the slow accumulation of fluid generally prevents tamponade. Thyroid hormone replacement effectively resolves these effusions over time, often without invasive procedures, highlighting the importance of early diagnosis and prompt management in preventing the severe cardiopulmonary complications of myxedema coma.

In advanced and severe stages of hypothyroidism, approximately one-third of patients can present with a pericardial effusion [7,8]. This occurs due to an increase in vascular permeability, allowing fluid to enter the interstitial space. In most cases, pericardial effusion can lead to cardiac tamponade; however, in hypothyroidism-related pericardial effusion, the accumulation of fluid is slow, preventing a tamponade from arising [9]. Thyroid replacement therapy has been shown to be an effective treatment for these effusions with complete resolution within months to years once the euthyroid state is reached, therefore not requiring pericardiocentesis to drain the effusions [8].

## Conclusions

Hypercapnic respiratory failure as a manifestation of severe myxedema coma is an exceptionally rare but life-threatening presentation, most often occurring in patients with untreated or undiagnosed hypothyroidism. Early recognition and prompt intervention are critical to prevent progression and reduce mortality. Management with thyroid hormone replacement, primarily levothyroxine, remains the standard of care. In inpatient settings, additional supportive interventions, including warming measures, corticosteroids, and vasopressor therapy, may be necessary to stabilize patients and improve survival outcomes. Patients with acute respiratory compromise often necessitate supportive measures such as intubation and mechanical ventilation. Patient counseling on strict adherence to thyroid medication is essential for long-term prevention.
